# Inverted insulin to C-Peptide ratios in neonatal intensive care: is there something we don't know?

**DOI:** 10.3389/fped.2026.1900675

**Published:** 2026-08-05

**Authors:** J. Geoffrey Chase, Helen D. Shannon

**Affiliations:** 1 Department of Mechanical Engineering, Centre for Bioengineering, University of Canterbury, Christchurch, New Zealand; 2 OCTO, Cheshire, United Kingdom

**Keywords:** C-Peptide, I/C ratio, insulin, insulin poisoning, neonate, NICU, pre-term

## Abstract

**Background:**

Insulin (I) and C-Peptide (C) are secreted in equimolar amounts, but insulin clears more rapidly. Typical Insulin to C-Peptide ratios (I/C) are therefore <0.05–0.2 (1/20–1/5). Except in relatively rare cases, I/C > 1.0 are interpreted as evidence of exogenous or factitious insulin dosing. However, recently published data from pre-term neonates with no insulin dosing or family history of diabetes show far higher ratios. How can these “*inverted*” ratios happen without factitious insulin poisoning?

**Hypothesis:**

Insulin binding antibodies explain the incidence and magnitude of “inverted” I/C ratios in this NICU cohort?

**Method:**

A secondary analysis integrating three independent evidence streams with mechanistic modelling study. Three “siloed” areas of neonatal research were integrated: (1) paired insulin and C-Peptide measurements from two NICU cohorts (227 and 75 matched samples) from infants not receiving insulin and with no immediate family history of diabetes or insulin use illustrates the incidence of unexpected I/C > 0.1–0.2; (2) Analysis of direct measurements of affinity, displacement and binding-site capacity across 5 independent studies constrain neonatal binding affinity and concentration; and (3) Analysis of binding kinetics of reversible insulin-binding reactions. Results of #2–3 are combined with a clinically validated neonatal metabolic model to assess whether they could account for observed I/C > 1.0 from #1.

**Results:**

For #1, over 80% of NICU infants had I/C > 0.1 and 40% had I/C > 1.0 consistent with circulating bound insulin measured by some immunoassays. #2, Independent affinity measurements, displacement studies, direct concentration measurements, and reconstruction from I/C data converged on a dominant high-affinity, low-nanomolar binding regime. #3, Model-based analysis of antibody binding reactions reproduced the observed I/C ratios and high apparent insulin concentrations without requiring extreme assumptions.

**Conclusion:**

High I/C ratios are not uncommon in NICU neonates and can be explained by high-affinity insulin-binding activity rather than insulin administration. Multiple independent quantitative analyses converge on a common high-affinity, low-nanomolar binding regime which reproduces observed I/C ratios. Critical illness, such as sepsis, significantly increases the levels and likelihood of unexpected high I/C ratios.

## 1 Introduction

Insulin (I) and C-Peptide (C) are secreted in equimolar amounts, but insulin clears faster via more pathways (1, 2). Thus, typical Insulin to C-Peptide ratios (I/C) are I/C < 0.05–0.20. These thresholds are well-established in pediatric and adult populations, with ratios of 1-to-5 to 1-to-15 noted (e.g., 3, 4), where I/C > 1.0, have been used to indicate exogenous administration or factitious insulin poisoning.

However, recently published results on unrelated topics presented paired insulin and C-Peptide samples, with many I/C ratios exceeding these thresholds (5, 6). The infants studied had no immediate family history of diabetes or insulin use and were premature and in the neonatal intensive care unit, but not otherwise critically ill. The results suggest a knowledge gap between pre-term neonates shortly after birth and healthy pediatric or adult subjects.

Very high insulin levels and I/C ratios can (rarely) occur in adults due to antibodies (7). In neonates, these syndromes/diseases are rare (8), while transient hyperinsulinism affects up to 50% of at-risk infants (9–11). However, C-Peptide samples in (5, 6) were in typical reported ranges (12–14), excluding hyperinsulinism as the primary cause. Further, even with zero hepatic clearance and insulin degradation, insulin is cleared up to ∼1.4× faster than C-Peptide by the kidneys alone (2). Thus, hyperinsulinism cannot cause I/C > 0.7–1.0 without a mechanism which markedly slows insulin clearance.

The study integrates published clinical datasets, published experimental measurements of neonatal insulin binding across 5 independent neonatal studies (15–19) (Supplementary Appendix A) and mechanistic modelling to examine whether insulin-binding antibodies can quantitatively demonstrate this phenomenon. It integrates directly measured paired neonatal I/C data (5, 6), direct measurements of neonatal insulin-binding activity, experimental affinity studies, known physics-based quantitative analysis of reversible insulin-binding reactions, and clinically validated neonatal metabolic modeling. Although these research areas are often quite “siloed”, their combined analysis assesses whether neonatal insulin-binding activity can account for independently observed, inverted I/C ratios.

## 2 Methods

This analysis and mechanistic modelling study combines neonatal datasets, direct measurements of neonatal insulin-binding activity, direct measurements of affinity, and proven displacement analyses and binding kinetics to quantitatively demonstrate how insulin-binding activity could yield the observed I/C ratios in (5, 6).

### 2.1 Paired insulin and C-Peptide NICU data

For clinical and ethical reasons, neonatal datasets providing paired C-Peptide and insulin measurements are very rare. Published data from healthy pre-term neonates without insulin treatment is available (5, 6). I/C ratios were reported in natural-log but not analyzed specifically. Data from Figures 5.1, 6.6–6.9 and Appendix 7 from (5) created two cohorts.

- Cohort 1: 102 non-insulin-treated euglycemic neonates: (67 male, 35 female; mean (SD) PMA 33.7 (4.36) weeks and mean birth weight of 2,071 (932) g.
- Cohort 2: 20 non-insulin treated hyperglycemic neonates from a separate study arm: (15 male, 5 female; mean (SD) PMA of 28.3 (1.52) weeks and mean (SD) birth weight of 1,109 (211) g.

Insulin and C-Peptide were measured using Invitron (Monmouth, UK) IV2-101 chemiluminescent kits with limits of detection 1.2 and 5.0 pmol/L, respectively. Sample times were not recorded explicitly, where (5, 6) state the majority of samples were taken within the first week of life and all within the first month. Table 1 reproduces the demographics. Complete details are in (5, 6). I/C ratios are presented as cumulative distribution functions (CDFs), reporting percentage of I/C ≤ 0.2, and I/C > 1.0, 4.0, and 10.0. Pairing insulin and C-Peptide values in this data shows the higher I/C ratios occur primarily at lower secretion rates (lower C-Peptide) and thus relatively very low free insulin. Applying the established physicochemical principles in the law of mass action to these data strongly constrains the affinity of neonatal insulin binding in healthy neonates to KD ∼ ≤ 1 × 10^−10^ M (Supplementary Appendix A). Cohorts were analysed separately as they likely had differing levels of critical illness (20–22).

**Table 1:** Demographic data reproduced from (5, 6).

Measure	Cohort 1 (SD, *n*)	Cohort 2 (SD, *n*)
Gestational age (weeks)	32.7 (4.2, 102)	27.9 (1.4, 20)
Postmenstrual age (weeks)	33.7 (4.4, 102)	28.3 (1.5, 20)
Birth weight (g)	2,071 (932, 102)	1,109 (211, 20)
Weight (g)	2,055 (871, 78)	1,110 (277, 17)
Length (cm)	43.3 (5.7, 93)	37.2 (3.6, 17)
Head circumference (cm)	30.3 (3.8, 94)	26.0 (1.7, 18)

### 2.2 Direct measurement of insulin-binding site concentration in neonates

Insulin bound to antibodies is measured as insulin in many immunoassays. Consequently, insulin-binding activity (IA) effectively “stores” insulin in blood up to several months, removing it from normal insulin clearance pathways. The result is high assay-measured insulin levels and I/C ratios (23–31). After accounting for differing assay specificity for IgG-mediated binding, 30%–95% of infants experience non-IgG insulin-binding, independent of prior insulin administration or immediate family history of diabetes (15, 17, 32, 33). Variation in reported prevalence is consistent with differing assay sensitivities applied to a continuous low-level binding distribution. This insulin-binding activity is induced by pregnancy (32). Although maternal IgG antibodies with insulin-binding capacity can cross the placenta (8, 34–36), the predominant neonatal binding activity identified in these studies was non-IgG, and the assays employed would not detect low concentrations of IgG IAs as found in 88% of adults (33).

**Figure 1:**
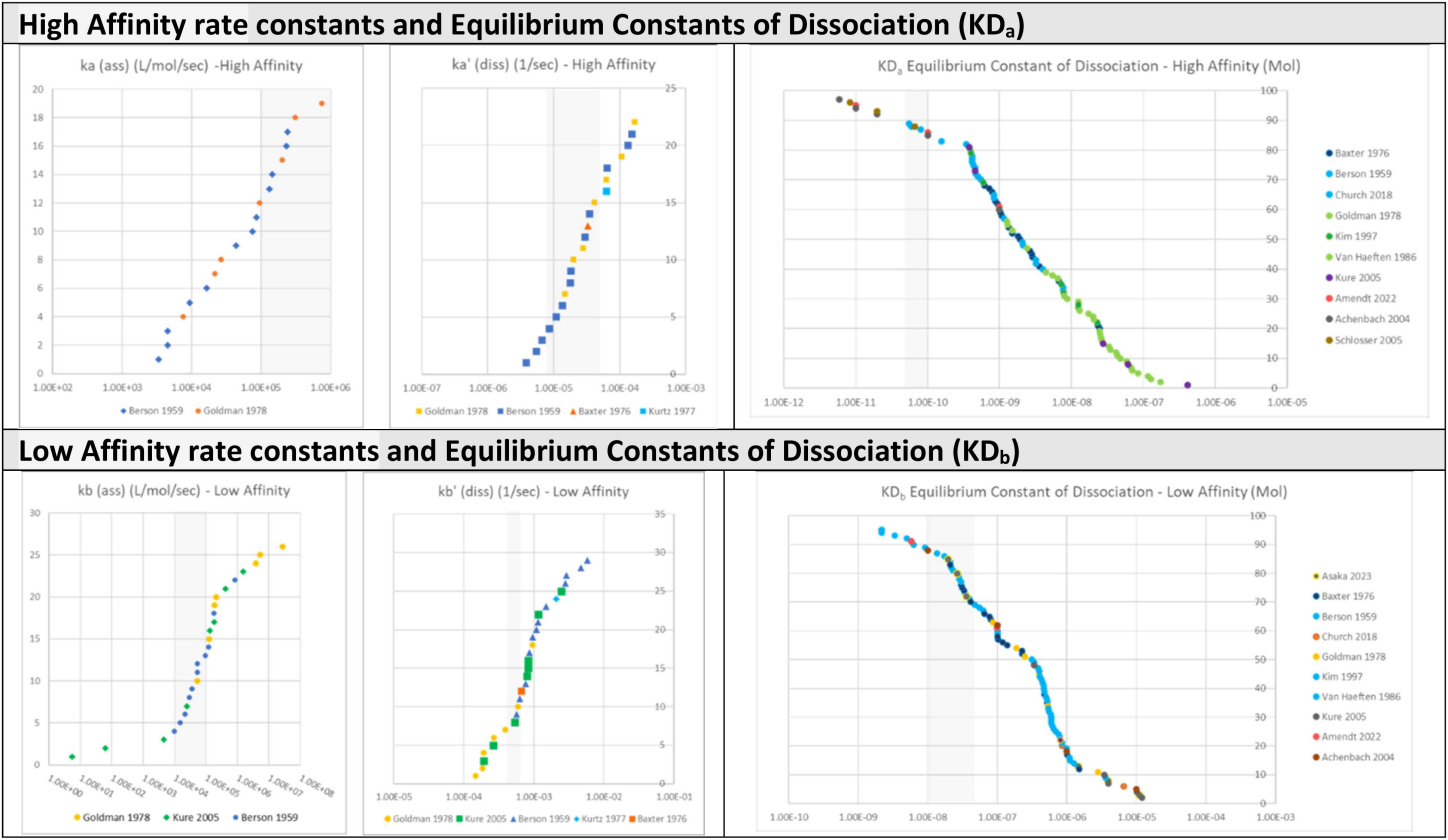
Graphical summary to show likelihood of a given rate constant for binding constants and dissociation constants, where ka, ka’ and kb, kb’ are the rate constants of association and dissociation for high affinity and low affinity binding respectively and K_D_ is equilibrium coefficients of dissociation. KD ranging (1 × 10^−7^, 8 × 10^−12^ Mol/L). Data are from (37, 50–54, 57, 60–63, 69, 70). Grey shading denotes simulation range.

**Figure 2:**
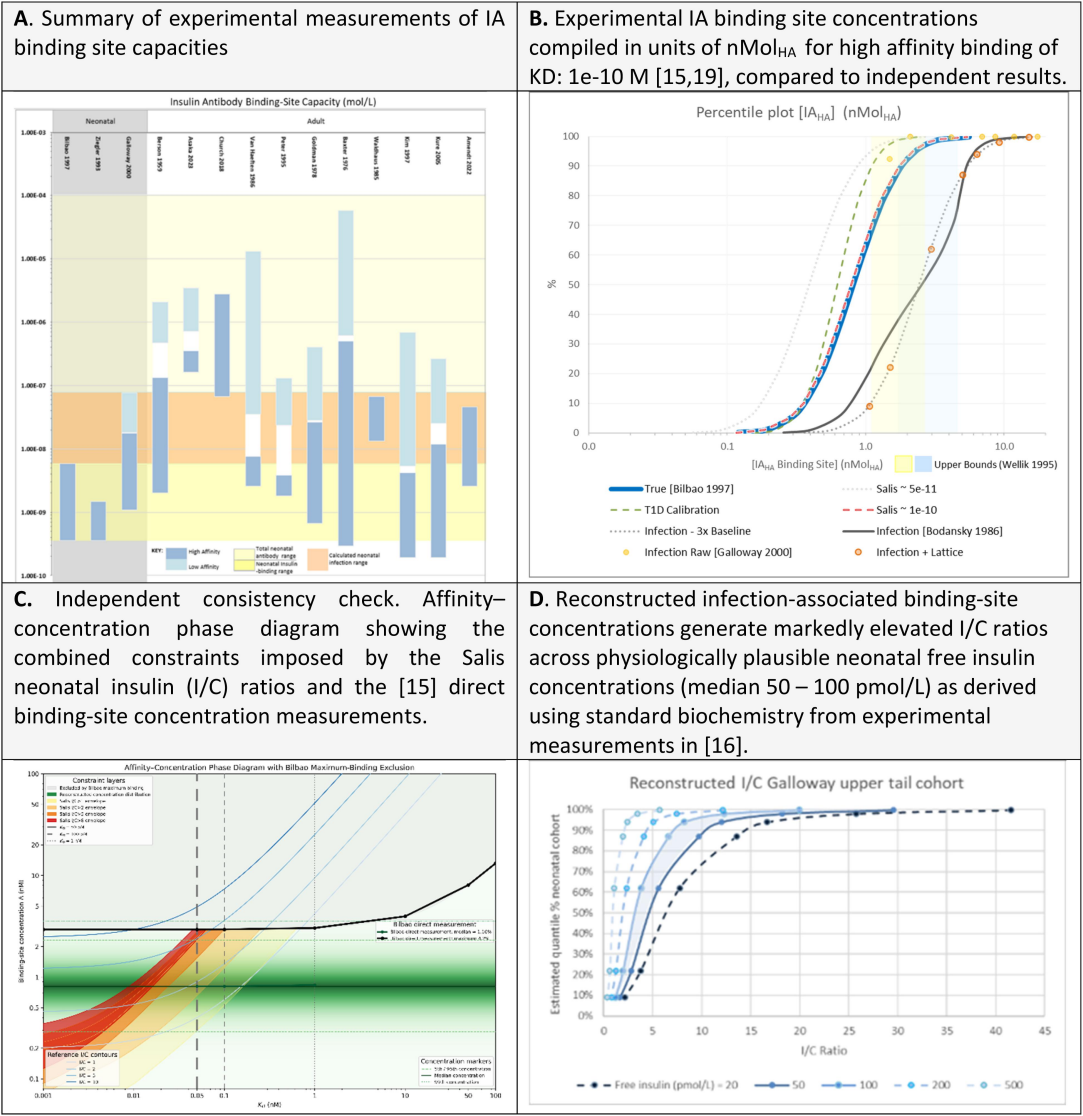
Integration of independent experimental measurements constraining neonatal insulin-binding affinity and concentration (15–19, 37, 50–55, 57, 58, 60, 69) with details in Supplementary Appendix A. **(A)** Shows insulin binding capacities reported across all studies. **(B)** Shows distribution of neonatal insulin binding sites normalised for affinity of KD = 1 × 10^−10^ M compared to independent results. **(C)** Shows the envelope of affinity and binding site concentration consistent with observed I/C ratios from (5, 6) as constrained by direct measurement values from (15). **(D)** Reconstructs I/C envelope from direct measurement (16) across an assumed neonatal free-insulin range.

Experimental measurement of neonatal insulin-binding activity has historically been reported in assay-specific units of nU/mL or binding % (15–19). Figures 1 and 2 show the experimentally measured data used in this analysis. Supplementary Appendix A integrates affinity constraints from Section 2.1 with assay design, affinity measurements, displacement studies and I/C reconstruction and identifies 6 independent quantitative constraints based on established physiochemical principles which converge on a dominant high-affinity binding process with binding-site concentrations in the low nanomolar range (Figures 2A,B). The experimentally observed affinity, complexing behaviour, and insulin-retention kinetics in these studies are compatible with a high-affinity antibody-mediated binding, including the possibility of high-affinity IgM (37).

Constraints on binding affinity and binding agent concentration derived from direct measurement of I/C (5, 6) are shown as bounded areas in Figure 2C up to the maximum measured binding site concentration (15), where both studies measured very similar cohorts. Overall, I/C > 2 is only consistent with a high affinity regime for binding site concentrations up to the maximum binding site concentrations measured in other studies in neonatal cohorts. The resulting experimentally constrained parameter ranges were then used as inputs to the mechanistic model.

Throughout, concentration calibration is normalised to a reference binding-site affinity of $K_{D,\mathrm{Site}}=10^{-10}M$, while lower measured effective affinities $\left(K_{D,\mathrm{eff}}\right)$ are interpreted as arising from multivalent avidity and complexing effects. The resultant normalised binding site concentration curve is used to assess the likelihood of IgG, IgM antibody or other agent binding site levels for modelling their impact on I/C ratios.

In addition to baseline neonatal IA, amplification can occur through infection, sepsis, antibiotics, steroids, antihypertensives, antioxidants and other pregnancy-related therapies (31, 34–36, 40–45), which is treated as a multiplier, as detailed in Supplementary Appendix A. Infection increases the concentration of maternal antibody transfer (16, 38) and oxidative stress, can enhance insulin-binding (37), and in neonates can also produce limited IgM (16, 38) and (later) IgG responses. Thus, non-critically ill neonates can experience amplification, although most research focuses on type 1 diabetes, where antibody binding is more common. While these insulin-binding antibodies are sometimes termed insulin autoimmune antibodies (IAA), there is no evidence neonatal triggers are uniformly auto-immune, so “IA” is used throughout to denote insulin-binding activity generically.

Critically, some general insulin immunoassays also measure some or nearly all bound insulin (39–45), including 2-site immunoassays (44) used in (5, 6). Laboratory guidance recommends either antibody removal with repeat testing or forensic-grade LC-MS analysis when antibody interference is suspected (46–49). The result is total insulin measured can include unbound or free insulin, as well as relatively much higher amounts of bound insulin as it clears far more slowly than free insulin, resulting in high I/C ratios.

### 2.3 Binding reaction rates for insulin-binding antibodies and modeling

A brief literature survey shows binding (association) rates and unbinding (dissociation) rates for both low- and high- affinity insulin-binding antibodies are well-established (7, 16, 30, 37, 50–61), as are the underlying models (52, 59). These studies and models demonstrate substantial inter-subject variability, leading to a wide range of bound to unbound (free-to-act) insulin ratios.

Analysis based on direct measurements (16) and indirect reconstruction of ratios in (5, 6) indicate neonatal binding reactions exhibit very high affinity with direct measurement of effective dissociation equilibrium constant (KD_eff_) of ∼5 × 10^−11^ Mol. Reported affinities in literature span 1 × 10^−10^–6 × 10^−12^ Mol, including direct demonstration of high-affinity IgM in immature immune contexts (37) and mature IgG autoantibodies in childhood diabetes progression (61–63). Full details are in Supplementary Appendix A.

For modelling, two simultaneous single-site reversible binding reactions are used (52) for high-affinity (*a*) and low-affinity (*b*) binding:

$$[Ag]+[Ab_{a}]\begin{array}{c} ka\\ ⇌\\ ka^{\prime } \end{array}[\bar{AgAb_{a}}]\;KD_{a}=ka^{\prime }/\mathrm{ka}$$ (1a)

$$[Ag]+[Ab_{b}]\begin{array}{c} kb\\ ⇌\\ kb^{\prime } \end{array}[\bar{AgAb_{b}}]\;KD_{b}=kb^{\prime }/\mathrm{kb}$$ (1b)

Equilibrium and rate constants are summarised graphically in Figure 1 and Supplementary Appendix A. Figure 2 summarises insulin binding site concentrations relative to a distribution of neonatal values. Infection is modeled as increasing binding capacity from 2 to 4× (16, 38) with details in Supplementary Appendix A.

The goal is to determine whether the observed very high I/C ratios in (5, 6) can be reproduced using clinically validated neonatal insulin secretion (13, 14) and insulin-glucose metabolic models (64–66). Equation 1 are an established model to simulate bound and unbound insulin concentrations and with metabolic and secretion models yield immunoassay measured I/C ratios across neonatal affinity and concentration ranges in Figures 1 and 2. Antibody concentrations ranged from KD ∼1 × 10^−10^ to 5 × 10^−11^ M (Figure 2) for measured neonatal binding affinities and consistent binding constants were used (Figures 1, 2). To the authors’ knowledge, this dataset and modelling have not previously been integrated into a single analysis from antibodies and binding to metabolic results and resulting I/C ratios for bound and unbound insulin.

### 2.4 Analyses

I/C data from (5, 6) are presented as CDFs and percentages for I/C < 0.2 and I/C > 1.0, 4.0, and 10.0, with median, IQR, and minimum and maximum values to summarise the prevalence of elevated ratios in relation to the potential prevalence of insulin-binding antibodies. The model of Equation 1 and Figures 1 and 2 is simulated for 4-days to illustrate the possible range of I/C levels, using a clinically validated insulin and C-Peptide model for neonates (13, 14, 64–68). IA concentrations were selected from Figure 2B, including infection-associated (2–3×) IA increase (38). The results are placed in the context of the data from (5, 6). Combined, these two analyses show the very wide range of possibilities and should answer the hypothesis.

## 3 Results

### 3.1 Incidence: results of paired insulin and C-Peptide NICU data

**Figure 3:**
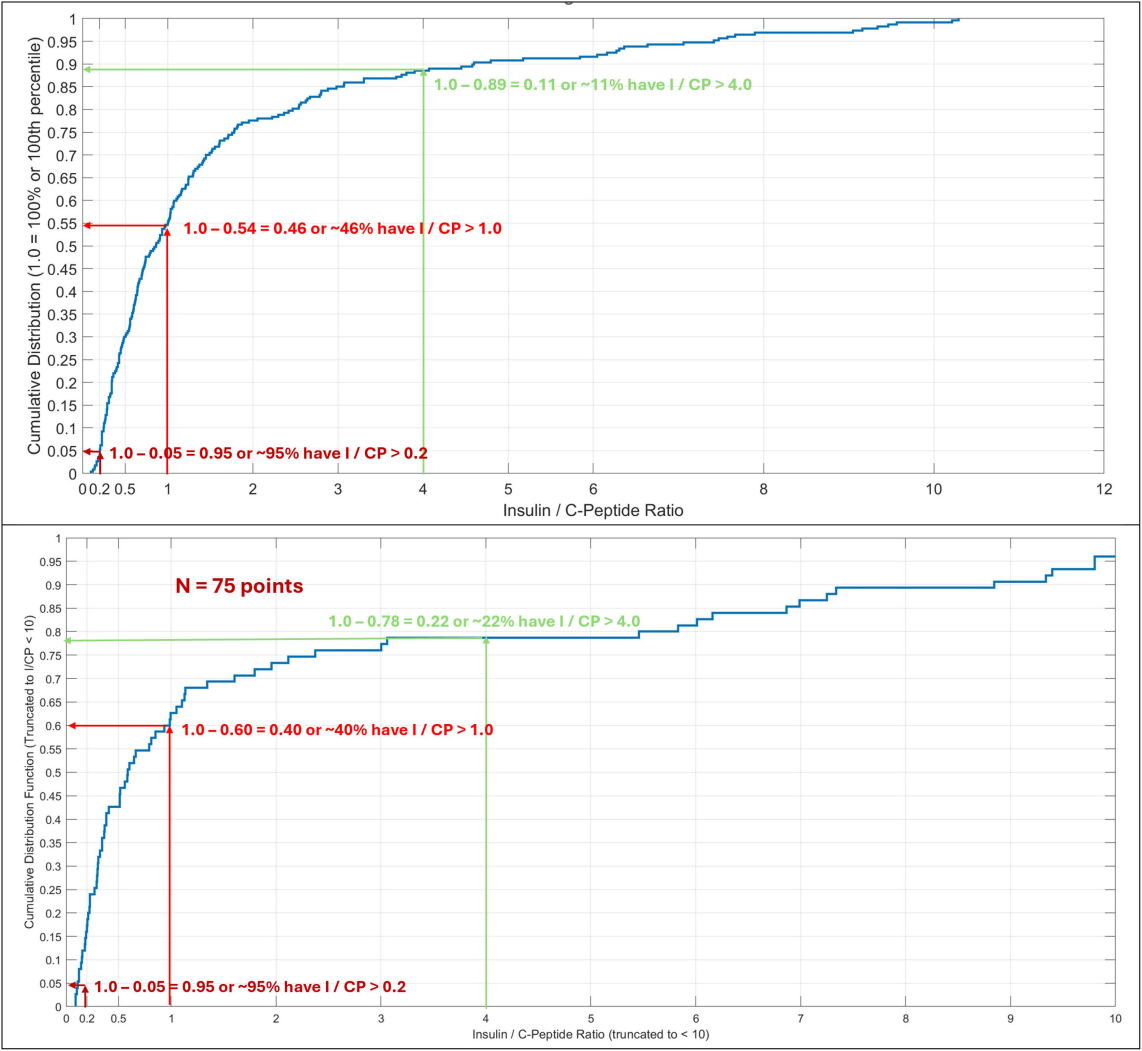
CDF of I/C ratios for euglycemic Cohort 1 (top) and hyperglycemic Cohort 2 (bottom). Neither cohort received insulin. Note I/C > 10.0 not included for clarity in Cohort 2 (max is I/C = 61.8).

Figure 3 presents I/C ratios for Cohorts 1–2. In Cohort 1 ∼5% of 227 matched pairs have I/C < 0.2, the commonly held upper limit. About 45% have I/C > 1.0 (insulin equals or exceeds C-Peptide), ∼11% have I/C > 4.0 and ∼0.9% exceed 10.0. Summary values are: (median; [IQR]; (max, min)) = (0.86; [0.43, 1.77]; (0.10, 10.29)). In Cohort 2 ∼5% of 75 matched pairs have I/C ≤ 0.2. Approximately 40% have I/C > 1.0, ∼22% have I/C > 4.0 and ∼1.0% exceed 10.0. Summary values are: (median; [IQR]; (max, min)) = (0.59; [0.28, 2.24]; (0.09, 61.8)).

### 3.2 Impact of antibody binding: modeling results

**Figure 4:**
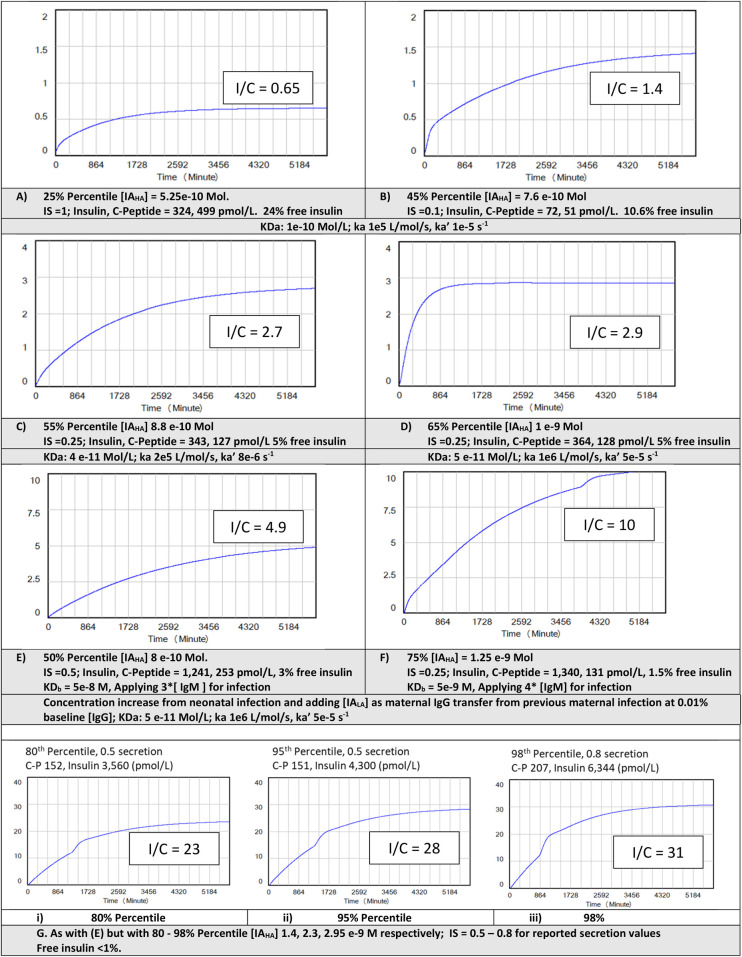
Time-averaged I/C ratios for up to 4 days for a range of example cases **A–G** using validated metabolic models (64–68), with IA_HA_ antibody titres which increase in infection **(E–G)**. IS = insulin secretion rate as a fraction of nominal rates for neonatal cohorts (13, 14). Binding affinity from Supplementary Appendix A and rate constants from ranges in Figure 1. These values lead to the resulting I/C ratio and insulin (bound and unbound) as would be measured by an immunoassay capturing both bound and unbound insulin and associated C-Peptide levels.

Figure 4 presents model results for a typical 1.5 kg neonate with median insulin sensitivity (71), typical nutrition and glucose demand (68), and insulin-binding parameters (KD, ka, ka') from Figure 1. Within the experimentally constrained affinity range (KD ≈ 5 × 10^−11^–10^−10^ M), model behaviour is driven predominantly by binding-site concentration, while alternative ka/koff combinations producing the same KD have relatively little influence. The dominant influence on bound insulin is Insulin binding site concentration, selected for IgM (15, 17) or an otherwise unconfirmed identity (18, 19) across mid-range values typical for newborns from Figure 2B. The insulin values noted include antibody-bound and free insulin, as would be seen by the assays used in (5, 6). Figure 4 also shows when high-affinity antibodies are present I/C could far exceed expected limits and the 80th percentile of Figure 3. In particular, the presence of maternal or neonatal infection (or both), combined with mid-range rate constants and affinities as measured experimentally for infected neonatal cohort (16), could yield very high I/C up to 30. These results are (Figure 2D) consistent with the experimentally measured extreme insulin binding measurements in the infected cohort of (16) showing how infection could increase these values.

These results are obtained with relatively low 10–50th percentile insulin secretion rates from (14, 67), resulting in C-Peptide levels of 60–444 pmol/L, matching (5, 6). Insulin levels span 98–6,340 pmol/L (typical-extreme), showing bound, un-cleared insulin could generate nearly any I/C value in Figure 3, over the 4 days simulated, using typical model parameters, and typical insulin secretion, antibody levels, and binding kinetics. Some values can be reached within 1–2 days (1,440–2,880 min), and more than 4 days would thus yield higher values for these cases. Insulin binding and metabolic modeling details and supporting data are given in Supplementary Appendix A.

## 4 Discussion

This study combines three independent sources of evidence: (1) direct clinical measurements of unexpectedly high I/C ratios in two neonatal cohorts (5, 6) and also independently observed as derived from the data from (16) as shown in Figure 2, (2) independent experimental measurements of neonatal insulin-binding site concentration and binding affinity constraints reported across and/or derived using established physiochemical principles from several studies and their data (5, 6, 15–19), and (3) clinically validated neonatal metabolic models (64–66) and etablished kinetic model physics to demonstrate the quantitative sufficiency of insulin binding to account for the observed insulin and C-Peptide clinical observations.

The hypothesis asks if neonatal insulin binding, whether antibodies or unknown protein, can quantitatively demonstrate the incidence and magnitude of “inverted” I/C ratios found in NICU cohorts? The results of this analysis quantitatively demonstrate the phenomenon directly observed in paired neonatal I/C data (5, 6) could be reconstructed from insulin binding signals independently measured in (16). Independent experimentally measured affinity, concentration, displacement, and calibration constraints (Supplementary Appendix A) converge on a high-affinity, low-nanomolar binding regime using established physiochemical principles. Incorporated into clinically validated models of pre-term metabolism (64–66), this regime can reproduce the I/C ratios in (5, 6) and (16), without extreme assumptions for model parameters, antibody concentration, or binding affinity.

Thus, insulin-binding antibodies can provide a coherent explanation for I/C ratios otherwise viewed as suggestive of exogenous or factitious insulin. No prior studies establish normative I/C ratios for this cohort, so this work highlights both the “curious” inverted I/C ratios and provides a potential physiological explanation. Importantly, key studies enabling this analysis were published relatively recently in 2016–2023 (5, 6, 37, 72). Hence, antibody-mediated insulin binding in the NICU cohort remains incompletely recognised/understood in the literature.

Figure 3 shows for non-insulin-exposed euglycemic (top, *N* = 227) and hyperglycemic (bottom, *N* = 75) infants, ∼95% had I/C > 0.2 with 40%–45% being I/C > 1.0. Maximum values of 10 and 61, respectively, were observed. These data indicate the phenomenon of elevated to inverted I/C ratios may affect a large proportion of NICU infants, consistent with widespread insulin-binding activity in neonatal studies (Figures 1, 2) and (5, 6).

Figure 3 also shows applying a high-affinity low-nanomolar binding regime to Equation 1, can reproduce I/C ratios seen in (5, 6) for modelled affinities well within the range of direct measurements in Figure 2 (37, 61). High apparent insulin levels arise because antibody-bound insulin clears very slowly (23–31), producing, for typical secretion levels, very high I/C ratios over time in assays unable to distinguish antibody-bound from free insulin (39–44).

Figure 4 shows increasing percentiles of I/C results in (5, 6) to show the ability of natural neonatal physiology to produce, with these antibodies, the I/C ratios seen experimentally. In terms of sensitivity, affinities below 1 × 10^−10^ M show limited/negligible effect. However, this lack of sensitivity can also be determined biochemically applying the established physiochemical law of mass action, as illustrated in Supplementary Appendix A. The insulin secretion factor simulates the effect of variable regulation of insulin secretion. The effect of modest hyperinsulinism is not shown, but would only increase the overall levels of insulin and C-Peptide. Thus, the most dramatic influence on I/C ratio for a given binding site concentration is produced by limiting insulin secretion. This effect is predictable from the biochemistry in Supplementary Appendix A, where lowering insulin secretion so insulin concentration of ∼100 pmol maximises I/C ratio as it is near the value of KD. While affinity and secretion are the more sensitive parameters, this study has not examined all parameter combinations as it has a goal of showing the range of possibilities using non-extreme values for affinity and secretion.

Equally, transient hyperinsulinism can be common (9–11). Differences in clearance rates between insulin and C-Peptide mean hyperinsulinism by itself cannot cause high I/C values without a mechanism to dramatically reduce insulin clearance, such as antibody binding. However, insulin secretion is highly variable in these cohorts (13, 14, 67), and, combined with antibody binding, may increase the I/C values seen and/or accelerate its rate of increase, where Figure 4 assumed constant, typical levels of insulin secretion. Further, the C-Peptide values in (5, 6) were in reported ranges (12–14) removing hyperinsulinism as a cause. Thus, hyperinsulinism may be a factor, but cannot be the sole cause of inverted I/C values.

Finally, Figure 3 shows little difference between Cohorts 1 and 2. This outcome suggests hyperglycemia (Cohort 2) did not play a role in elevating I/C, further supporting the fact hyperinsulinism alone cannot create inverted I/C ratios due to first pass hepatic extraction and a greater number of clearance routes. In this sense, it provides support for antibody binding to insulin, or another agent, for creating the observed results in (5, 6) and (73). We would also note insulin secretion in pre-term neonates is highly variable and not strong correlated to glycemic level (13, 14).

Literature suggests several other potential triggers beyond pregnancy. The results here suggest insulin-binding activity may influence a larger proportion of NICU infants than recognised by less sensitive assays (24–28, 74). More critically ill infants, as potentially seen in Cohort 2, may also exhibit higher concentrations of bound insulin. Cohort 2 had a higher maximum I/C with a similar range, reflected in the skew in Figure 3 (bottom).

The analysis integrates several previously disconnected areas of neonatal research, including insulin-binding prevalence studies, affinity measurements, displacement analyses, concentration calibrations and I/C observations. While the available neonatal literature remains limited, these independent datasets show remarkable quantitative consistency, converging on a common high-affinity, low-nanomolar binding regime.

Together, these observations, quantitative analyses, and modelling results show unexpectedly high I/C ratios could be relatively common in NICU cohorts, and antibody-mediated insulin retention providea a credible explanation. Thus, unlike other cohorts of different ages not exposed to insulin, high I/C ratios in neonates should not be assumed indicative of exogenous insulin but instead require further investigation for confirmation. This outcome reinforces guidance stating discordant immunoassay I/C readings should prompt antibody testing and/or retesting using LC-MS or similar forensic-grade assays (46–49).

The main limitation of this study is the molecular identity of the neonatal binding agent remains uncertain. However, the underlying phenomenon is directly observed in contemporary neonatal cohorts through marked inversion of I/C ratios, while independent affinity measurements, displacement studies, direct concentration measurements, and multiple external constraints based on biochemistry and physics converge on the same high-affinity, low-nanomolar binding regime capable of explaining these observations.

There are other agents which can bind with insulin which can be found in critical care environments. These factors include soluble proteins with insulin-binding capability such as Alpha-2-macroglobulin (A2M) and circulating insulin receptor fragments generated through inflammatory receptor shedding, as well as potentially other insulin-binding ligands. Based on current evidence, these factors generally appear to exhibit either affinity lower than the threshold of KD 1 × 10^−10^ M and/or lower effective binding-site concentrations and inconsistent clearance rates, and would thus not meet the combination of affinity and binding site concentration required. However, as such factors are not routinely measured in pre-term, critically ill, or other neonatal, cohorts, some uncertainty remains regarding the precise identity of the binding agent(s) contributing to the measurements in (5, 6). Currently, this uncertainty will ultimately require prospective study as there is no complete data offering a clearer picture than presented here.

In particular, the only molecule in our view that satisfies all available data is high affinity IgM. This preliminary conclusion is unproven prospectively. Thus, while IgM has been measured in neonates, this effect as presented/proposed has not yet been directly measured in neonates. As shown in Figures 1 and 2, Supplementary Appendix A, and the results, there are experimentally measured concentrations, affinities, and biochemistry constraints which indicate the regime proposed has potential. However, the last step, direct measurement of antibody bound insulin with high I/C has not been shown. That said, the results of (16) offer an inference for future research to explore as one interpretation satisfying all reported readings in their study is complexing of high affinity IgM from the inability of the IgG and IgM components to account for total binding. Hence, high affinity IgM would be a candidate with this supporting evidence as a first target of future research.

However, given other possibilities, such research should be highly specific about the assays used and their potential for interference with antibody bound insulin (which can vary by assay) or any other factors. The use of mass spectroscopy would allow more refined assessment of the components leading to measured I/C values to better identify potential other binding agents, but would require potentially greater blood sampling. Thus, without a prospective test, it is not possible to conclude the exact agent or agents, but the evidence available in support of antibody binding presents the best argument, in the authors' opinion, for the mechanism and does quantitatively demonstrate its potential for creating these observed I/C values.

A further limitation is reliance on direct I/C ratios from two related studies in a single NICU, though there is supporting evidence directly derived from the results in (16, 18) in Figure 2. The authors were able to find a very recent study with limited numbers of tests showing paired I/C samples (73). This study reported median I/C values of 0.42 and 0.35 at two time points with IQR values all above the adult expected values of 0.05–0.20, so they were elevated but not inverted, although the full range was not reported. This study used a BioPlex assay which may only recover a smaller portion of bound insulin than other assays, where this has only been studied on an older BioRad version (41). A higher recovery of bound insulin would lead to greater I/C all else equal. There is thus independent data this phenomenon exists, but such data is very limited because this population is fragile, meaning large-scale testing to establish normative values for insulin, C-Peptide, and/or I/C values is clinically and ethically difficult, and such datasets remain rare.

This added study (73) highlights three key outcomes with respect to this limitation. First, the type of assay matters and not all assays are equal in their crossover. Studies which do not use assays capturing bound and free insulin (39–45) would not see this phenomenon, and not every unit uses such assays. Second, higher than expected, if not inverted, I/C values have been observed in other studies. Third, further independent studies, using multiple assay types or forensic quality mass spectroscopy, would strengthen the generalisability of these findings and help establish normative insulin, C-Peptide, and I/C distributions in NICU populations.

Supplementary Appendix A demonstrates inverting I/C ratios at physiologically plausible neonatal insulin concentrations requires a dominant high-affinity binding process with KD 1 × 10^−10^–5 × 10^−11^ M. Few affinity studies involve neonates. Thus, while the values identified here are supported by multiple, independent studies, the lack of affinity studies in neonates is a limitation. However, KD of 1 × 10^−9^–8 × 10^−12^ Mol/L have been reported in settings where antibodies are expected, such as infants of diabetic parents (61, 63), encompassing the affinity range identified here (Figure 1).

Modeling shows the affinity and concentration ranges identified from quantitative analysis are sufficient to reproduce the observed I/C distributions using established metabolic and binding kinetics, particularly in the presence of infection. While neonatal metabolic models carry inherent uncertainty, those used here have supported glycemic control in Christchurch Women's Hospital for 15 years with excellent results (68).

A final point needs to address what clinical data or studies would be required to confirm the entire chain of physiological effects presented is valid. The hypothesis would be weakened if prospective studies demonstrated high I/C ratios in the absence of measurable high-affinity neonatal insulin binding or if high I/C ratios were measured by an assay or biochemical technique known to be incapable of measuring bound insulin. Similarly, it would be weakened if measurements incapable of measuring bound insulin could not find such elevated I/C ratios as found in (5, 6, 73). In all cases, such studies should use insulin measurement methods, such as mass spectroscopy, to differentiate bound and free insulin, and quantify their amounts.

Any studies should be large enough to include a spectrum of term and pre-term neonate patient condition and both sexes. Such studies would better quantify, across multiple units, and potentially multiple countries/ethnicities, how likelihood of the observed high and inverted I/C ratios. Measurement using mass spectroscopy would allow first identification of one or more binding agents and clear differentiation of free and bound insulin, enabling better calibration of the kinetics of these actions. However, such studies would not quantify the exact agents or the exact chain of events leading to these I/C ratios.

To better quantify the exact chain of events, new studies should focus both on measurement of binding effect and measurement of binding agent. However, this approach requires repeat biochemical experimentation. Direct measurements might identify alternative binding agents with sufficient affinity, binding-site concentration and insulin-retention kinetics to quantitatively account for the observed I/C ratios. This outcome would direct attention from antibodies to alternative agents. Thus, it would not alter the main hypothesis, but would offer alternative interpretations of binding avidity at higher binding site concentrations.

## 5 Conclusions

High insulin to C-Peptide (I/C) ratios may be common in NICU infants, with maximum values in a published dataset reaching 10 and 61, and 40%–45% of values exceeding I/C > 1.0, indicating insulin dynamics in NICU and pre-term cohorts may differ substantially from those expected in older children and adults. Independent affinity measurements, displacement studies, concentration calibrations, and analyses converge on a dominant high-affinity, low-nanomolar binding regime which can quantitatively explain the observed I/C ratios. Incorporated into clinically-validated neonatal metabolic models, this regime quantitatively demonstrates the observed measured insulin concentrations and observed I/C ratios can be reproduced without requiring extreme antibody concentrations or binding affinities. However, these results are based on limited paired I/C data and remain to be prospectively demonstrated.

The principal clinical implication of this work is, in contrast to older paediatric and adult cohorts, high I/C ratios in neonates should not automatically be interpreted as evidence of exogenous insulin administration but require further investigation instead. In particular, supported by multiple, previously disconnected research areas, antibody-mediated insulin binding can provide a potential, quantifiably demonstrated physiological explanation. While the molecular identity of the dominant neonatal binding agent remains an important objective for future study, the underlying phenomenon has been directly observed in the limited I/C data available.

In short, pre-term NICU neonates are not “small adults” and may frequently exhibit “inverted” I/C ratios with no prior insulin exposure. Hyperinsulinism alone cannot explain these ratios. Insulin binding to quantified binding agents, whether antibody or unknown protein, could provide a quantifiable explanation, with critical illness, such as sepsis, maternal treatments, insulin exposure, or immediate family diabetes history, likely to further increase the magnitude and likelihood.

## Data Availability

The original contributions presented in the study are included in the article/supplementary material, further inquiries can be directed to geoff.chase@canterbury.ac.nz.
