# Supplementary material for: Inverted insulin to C-Peptide ratios in neonatal intensive care: is there something we don't know?
Source: Front Pediatr. 2026 Aug 5;14:1900675. doi: 10.3389/fped.2026.1900675 (PMC13485906; doi:10.3389/fped.2026.1900675)
Supplement: Supplementary file 1 [file Supplementaryfile1.pdf]

Appendix A: Concentration and affinity profiles for neonatal insulin binding

This appendix presents the modelling and quantitative framework for the neonatal insulin binding concentration curve used in the binding model and presented in **Figure 2B** in the paper. It is a concentration distribution of neonatal insulin binding-sites expressed in nanomolar units, which could be expected in this cohort based on the I/C data presented in the article and referenced works.

Three sections develop this distribution from literature data, defined by area:

A1. Affinity and concentration constraints from independent datasets: Reconstruction of neonatal binding using standard equilibrium models converges on a high affinity regime, confirmed by reconstruction of the I/C data presented in the article. Results show IA binding site concentrations converge within the low-nanomolar range.

A2. Impact of Infection on neonatal insulin binding: Infection-associated amplification is best explained as multiplicative enhancement over an existing high-affinity baseline, with observed elevation factors from literature of 2-4x.

A3. Consolidation: These results are brought together to create the calibration curve presented in **Figure 2.B** in the paper.

The following Equations E.1-E.7 are used throughout Appendix A:

$A + L \rightleftharpoons AL,$	$K_D = \frac{[A][L]}{[AL]}.$	f = [L]/([L] + K_D)	$[AL] = [A]_{tot}\theta$
$f = \frac{A_{T,\text{assay}} + T + K_D - \sqrt{(A_{T,\text{assay}} + T + K_D)^2 - 4A_{T,\text{assay}}T}}{2T}$		[A] = K_D . f/(1-f)	$I_{\text{bound}} = [A]\frac{I_f}{K_{D,\text{eff}} + I_f}$
**E.1. Equilibrium framework for insulin binding:**			
$[A]_x = D\left[f_x L_T + K_{D,x}\frac{f_x}{1 - f_x}\right]$			
**E.2 Ligand excess concentration calibration**			
f_res = f_total − f_IgG − f_baseline<br>$[A]_x = F(f_x, K_{D,x}, [L]_0)$ where: $x \in (total, IgG, res, baseline)$		Low-affinity residual model: $K_{D,res} \gg K_{D,HA}$<br>High-affinity residual model: $K_{D,res} \sim K_{D,HA}$	
**E.3 Residual binding decomposition framework**			
Imeas = I_f + q[AL]		Imeas/C = (I_f + q[AL])/C	
$[AL] = \frac{([A]_{tot}+I_f+K_D)-\sqrt{([A]_{tot}+I_f+K_D)^2-4[A]_{tot}I_f}}{2}$ with key limiting condition $K_D \sim I_f$			
**E.4 Equilibrium reconstruction of I/C ratios**			
Bilbao to Marzinotto percentile mapping $Q_N(p) \sim Q_{T1D}(p)$ and $[A]_N = s \cdot B$<br>where: B= Bilbao binding signal, s= empirically derived scaling coefficient.<br>Affinity adjustment under the antibody-excess approximation.: $[A]_{HA} = [A]_{T1D}\frac{K_{D,T1D}}{K_{D,HA}}$			
**E.5 Cross-cohort calibration to early-onset T1D**			
$[A]_{nMHA} = [A]_x\frac{L + K_{D,HA}}{L + K_{D,x}}$		$\phi_{\text{lat}} = \phi_0 + (\phi_{\max} - \phi_0)\frac{[A]_{\text{lat}}^h}{A_{50}^h + [A]_{\text{lat}}^h}$	
**E.6 nM_{HA} scaling equation**		**E.7 Lattice-transition equation**	

[A]	binding-site concentration	(M)		I_f, I_{bound} I_{meas}	Free, bound and measured insulin concentration	(M)
$[A]_{lat}$,A_{50}	effective lattice concentration, half-maximal lattice-transition concentration	(M)		K_D or K_{DSite}, K_{Deff}	Site calibration dissociation constant, apparent / effective dissociation constant including avidity effects	(M)
AL	ligand–binding-site complex	(M)		ka’	reverse binding rate constant	s^{-1}
C	C-peptide concentration	(M)		L	free ligand / tracer insulin	(M)
D	assay dilution/scaling factor	dml		L_T	total tracer concentration	(M)
f_x,f_{res}	measured and residual binding fraction	dml		q	assay recovery factor for bound insulin	dml
h	Hill coefficient	dml		Q(p)	percentile function	%
ϕ_{lat}, ϕ_0, ϕ_{max}	Latticed fraction, baseline non-displaceable lattice fraction, maximal lattice fraction	dml		s	empirical cross-cohort scaling coefficient	dml

1. Affinity and concentration constraints from independent datasets

Published neonatal insulin-binding studies employed two complementary assay designs. First, ligand-limiting assays [16-18] are highly sensitive to binding affinity and therefore provide direct constraints on the strength of the binding reactions. Second, and in contrast, ligand-excess assays [15,19,74] operate under near-saturating tracer conditions, providing direct estimates of binding-site concentration largely independent of affinity across the high-affinity range.

Reanalysis of published displacement data [16] using standard equilibrium binding **Equations E.1**, **E.3** and Cheng Prusoff approximation yields a dominant high-affinity component KD_{eff}~ 5e-11 M and a less robustly defined low-affinity component KD_{eff} of order 1e-8 M (measured 3.8 e-8 M).

Direct displacement measurements and independent reconstruction from neonatal I/C ratios indicate a dominant high-affinity binding regime (KD ≤ 10^{-10} M). **Figure A1** shows key elements of this result.

Specifically, binding-site concentration was estimated by applying full equilibrium binding equations using six independent approaches:

- **(i)** direct calibration of ligand-excess assays [15,74] (**Equations E.1,E.2**) (**Figure A1.B**) supported independently by
- **(ii)** comparison with early-onset type 1 diabetes datasets as noted in [15] (**Equations E.5,E.6**) (**Figure A1.B**).
- **(iii)** reconstruction from neonatal I/C ratios ([5,6,12]) (**Figure A1.C**) (**Equation E.4**)
- **(iv)** Binding affinity is constrained directly by the profile of neonatal I/C ratios vs free insulin ([5,6]). Equilibrium binding equations (**Equation E.4**) yields **Equation A-2** below, which in turn reveals a unique property of insulin binding, specifically that a dramatic rise in I/C ratio is inevitable if free insulin concentrations are of similar magnitude to the effective dissociation constant. (**Figure A1.D(i,iii)**

$$\mathrm{I{:}C} = \frac{1}{5}\left(1 + \frac{A}{K_D + F}\right) \qquad \text{(A-2)}$$

Where *F* is free insulin (I_f) and *A* is binding site concentration of antibodies [A] or any agent which binds insulin.

- **(v)** When combined with direct calibration [15] (**Equation E.2**), the affinity-concentration phase diagram based on direct measurement of I/C [5,6] is constrained to the high affinity region (**Figure A1.E**). I/C contours (**Equation A-2**) represent the theoretical combinations of affinity and concentration. I/C data from Salis et al [5,6] are overlaid in coloured envelopes by applying **Equation A-2** across the affinity range, and clipped by the independent Bilbao maximum binding-site constraint.
- **(vi)** Physiological limits imposed by measured free insulin concentrations [18] (**Equations E.1 and E.4**) (**Figure A1.F**).

Despite differing methodologies and assumptions, all approaches converged on a common low-nanomolar binding-site concentration range with an approximately log-normal distribution.

The neonatal insulin-binding agent is consistently reported as non-IgG mediated [15,17,32,36,61,75-78] but otherwise molecular identity remains uncertain. Historical studies [15-17] have proposed both an unidentified binding protein and IgM-mediated binding. Conventional low-affinity IgM [79] is inconsistent with the observed binding behaviour (**Figures A1.D(ii),A1.E**), whereas recent studies demonstrate the existence of high-affinity IgM species [37,72]. Irrespective of uncertainty of identity, the quantitative analyses presented here are independent of binding species.

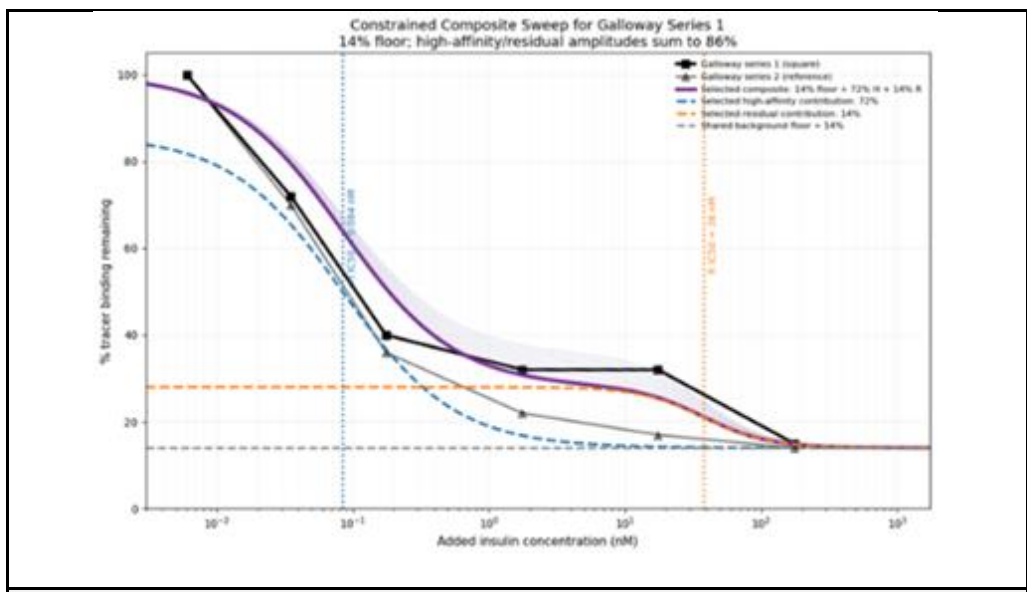

A. Reanalysis of Galloway et al [16] displacement data identifies a dominant high-affinity component (KD 5e-11 M).

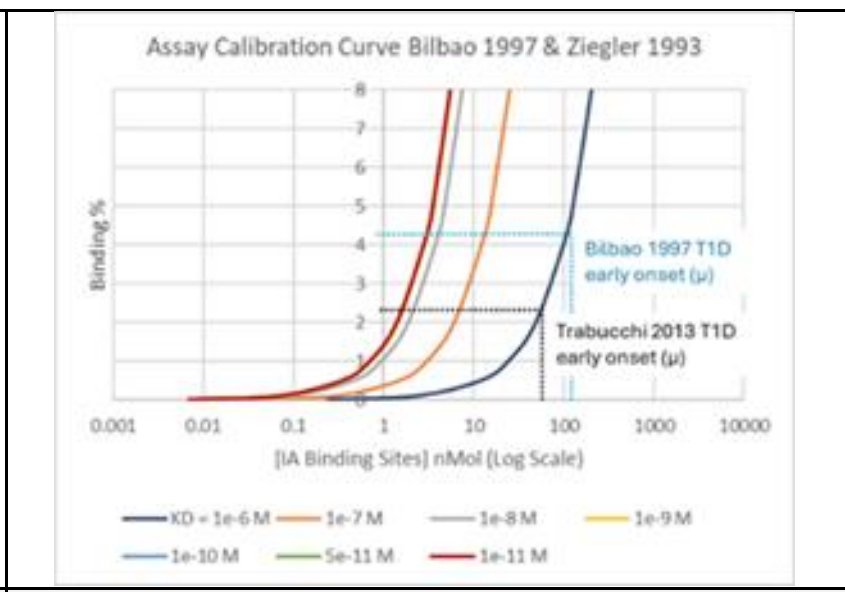

B Ligand-excess calibration [15] demonstrates that binding-site concentration is largely affinity-independent across the high-affinity range and provides benchmarking against early-onset T1D datasets [80].

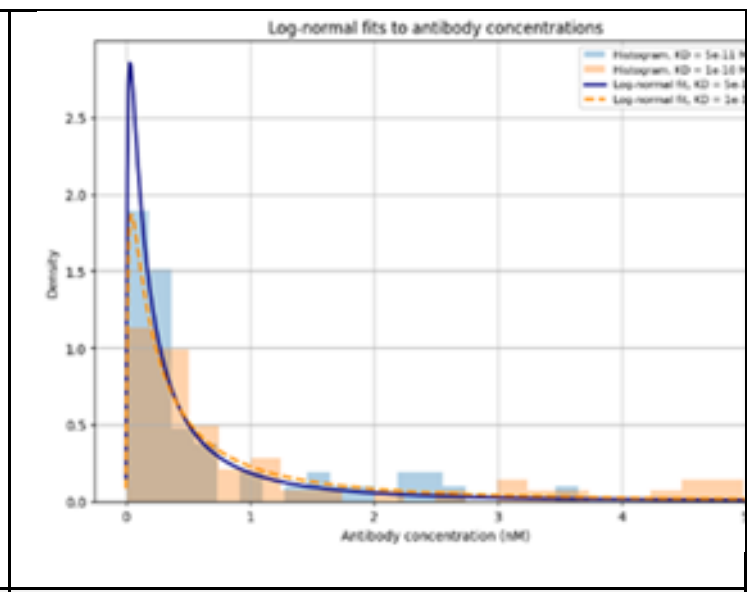

C. Reconstructed neonatal binding-site concentrations [5,6,12] show an approximately log-normal distribution in the low-nanomolar range under affinity assumptions K_{Dsite} range 1e-10 M (orange) to 5e-11 M (blue).

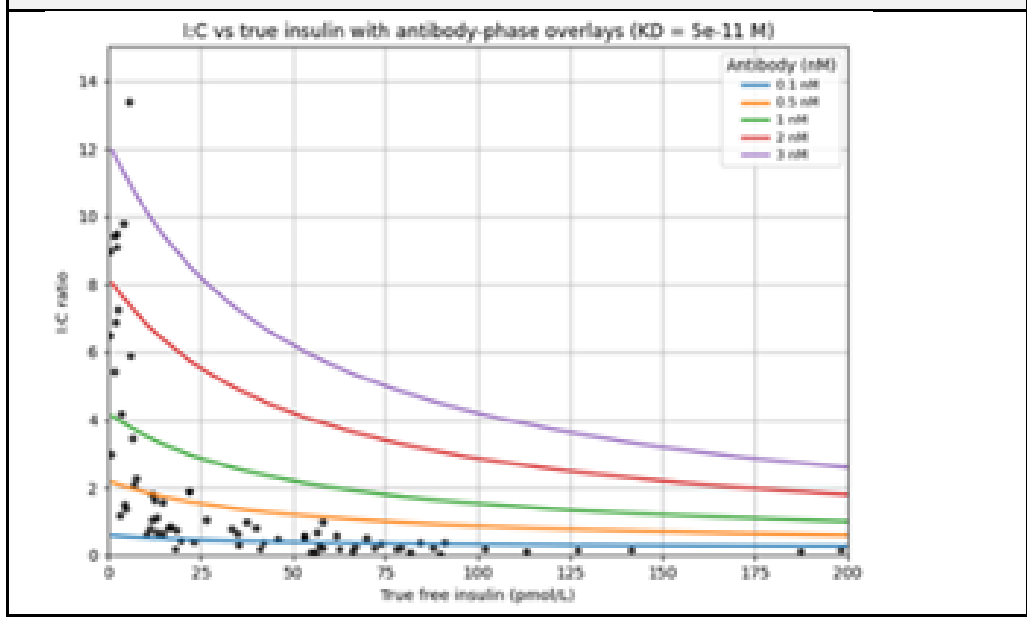

i

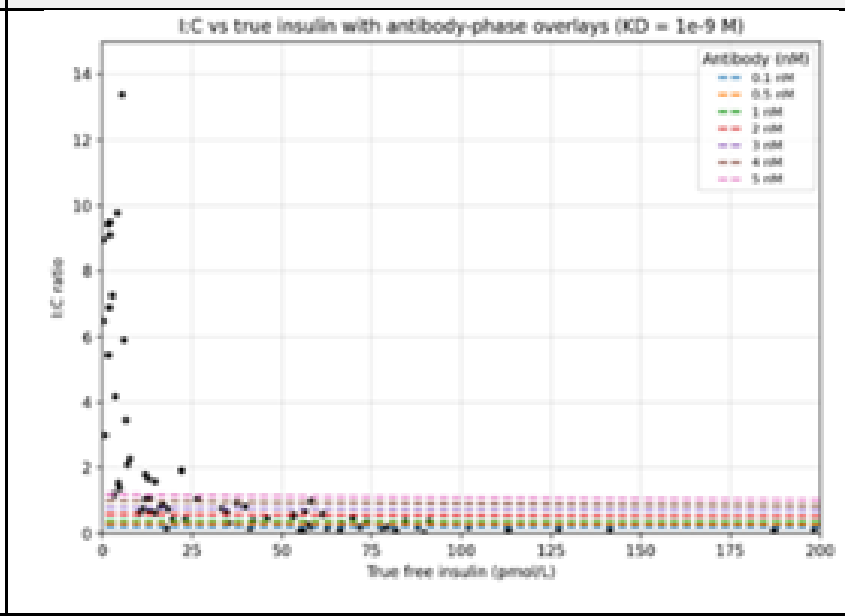

ii

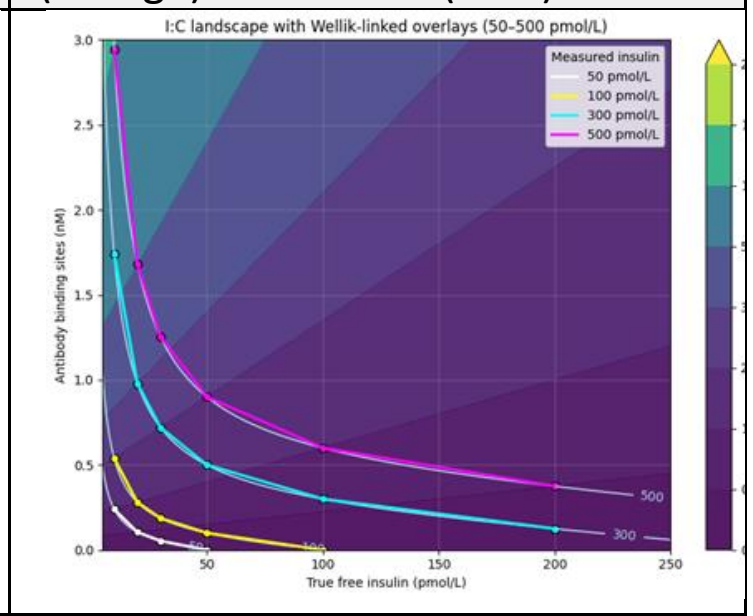

iii

D. Independent neonatal I/C ratio and free-insulin datasets converge on a high-affinity binding regime. Observed I/C ratios [5,6] (black dots) are reproduced under high-affinity conditions **(i)** but not lower-affinity conditions **(ii)**. Independent free-insulin measurements [18] occupy the same high-affinity solution space **(iii).**

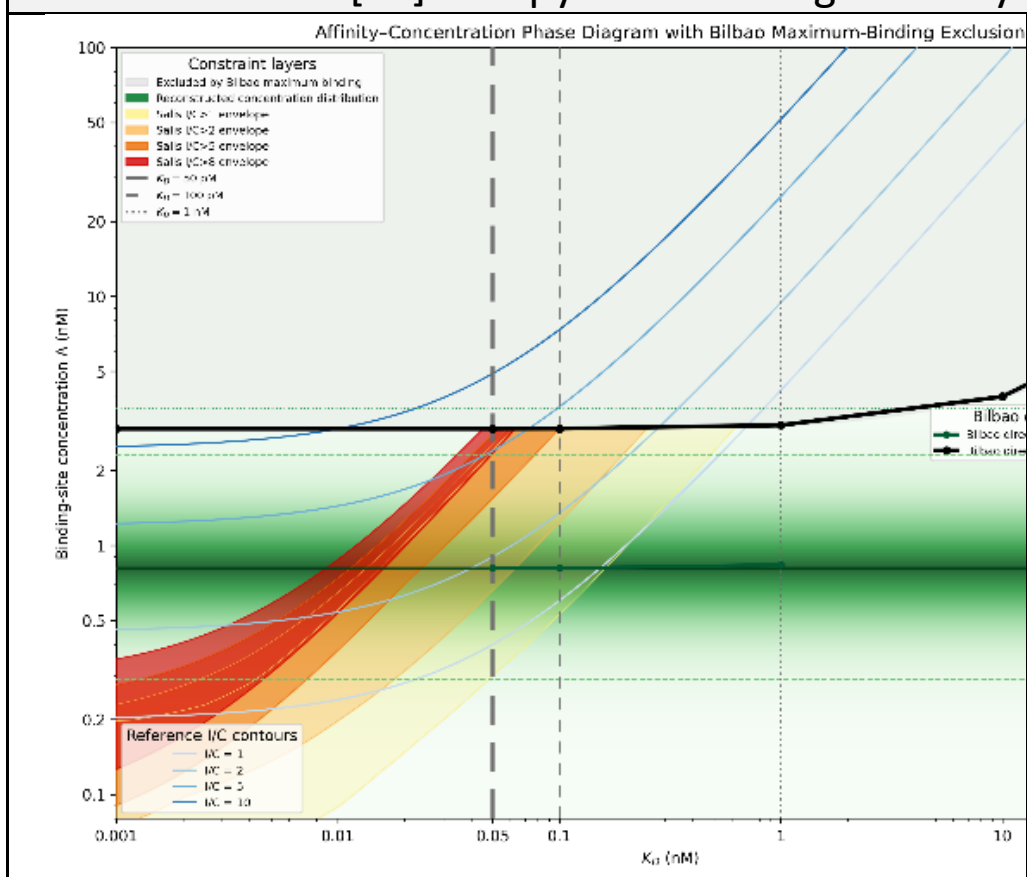

E. Constraints of neonatal insulin (I/C) ratios [5,6] are shown by the coloured envelopes and bounded by the maximum concentration permitted by the Bilbao direct binding measurements. Grey vertical lines indicate representative affinity values. Overlap of these constraints limits the affinity–concentration space for the Salis cohort to the high-affinity regime.

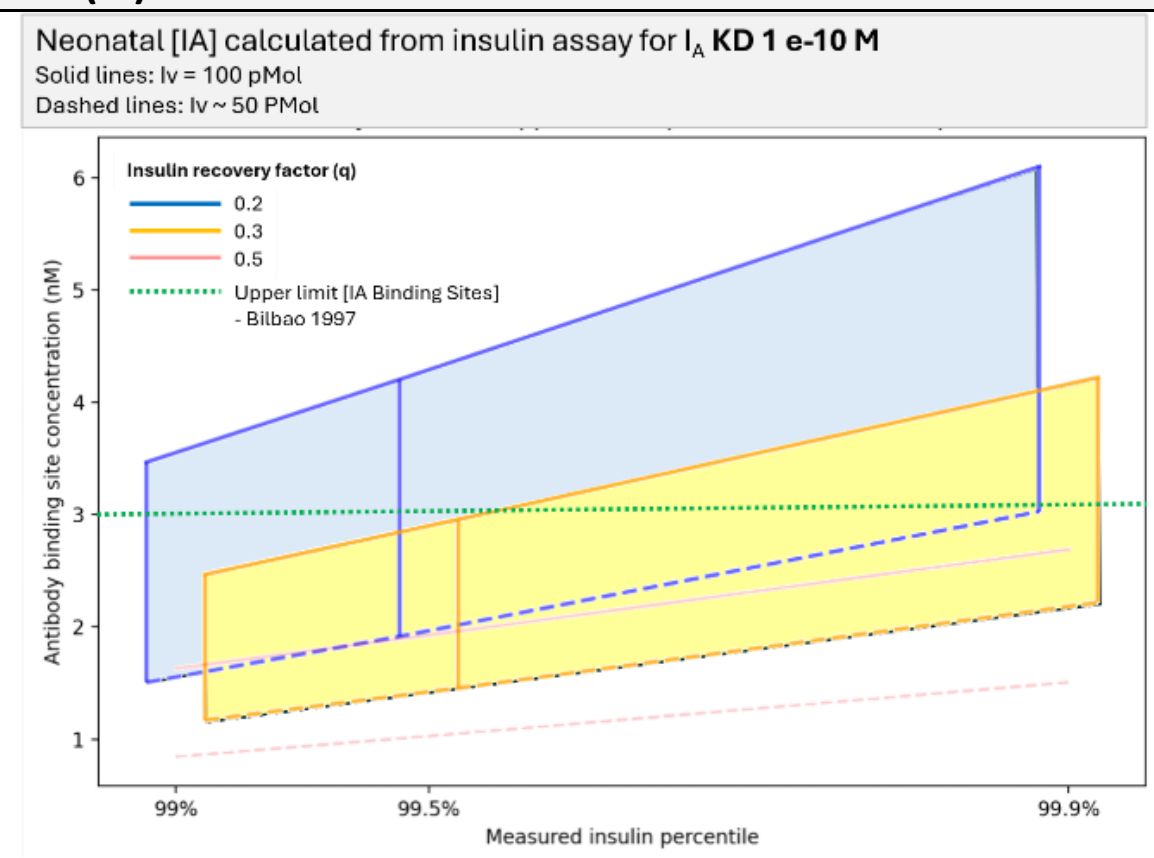

F. Range of maximum (99% to 99.9% percentile) limits of binding site concentration for assumed KD 1e-10 M, based on free insulin measurements [18].

Figure A1: Independent affinity and concentration constraints from neonatal insulin-binding datasets. Direct displacement analysis, neonatal I/C ratio reconstruction from [5,6], ligand excess calibration [15] and free-insulin measurements [18] converge on a high affinity, low-nanomolar binding regime with KD ≤ 10^{-10} M.

2. Impact of infection on neonatal insulin binding

Galloway et al. [16] identified a high-binding subgroup comprising ~7% of cord blood samples and interpreted it as consistent with an infection-associated effect. Both IgM and IgG binding were observed, although no specific pathogen was identified. Reconstruction of the cohort (**FigureA2.A**) showed elevated binding activity could not be explained by addition of a low-affinity component alone.

Instead, the observed binding profile required a substantial increase in high-affinity binding capacity, extending concentrations with an approximate threefold upward shift in binding site concentration from approximately 5–6 nM to around 15 nM. These findings are consistent with independent observations by Bodansky et al. [38], who reported 2-4x increases in insulin-binding antibodies during infection.

[16]further reported a substantial binding component in an infected cohort consistent with cooperative complex formation. Although the molecular identity of the neonatal binding agent is uncertain, multivalent high-affinity IgM [37,72]is a plausible explanation because it is capable of forming soluble insulin-linked complexes and lattice structures [79,81-84]. IgG showed weaker association with the residual component, suggesting it may reflect secondary involvement during infection rather than the primary driver of the effect.

For the purposes of this study, infection is represented as multiplicative amplification of an existing high-affinity baseline binding system. This infection-adjusted distribution is incorporated into the consolidated concentration profile used throughout the analysis. Overall, at these concentrations, equilibrium reconstruction predicts marked increases in total insulin and I/C ratio while maintaining physiologically plausible free insulin concentrations. (**Figure A2.B**)

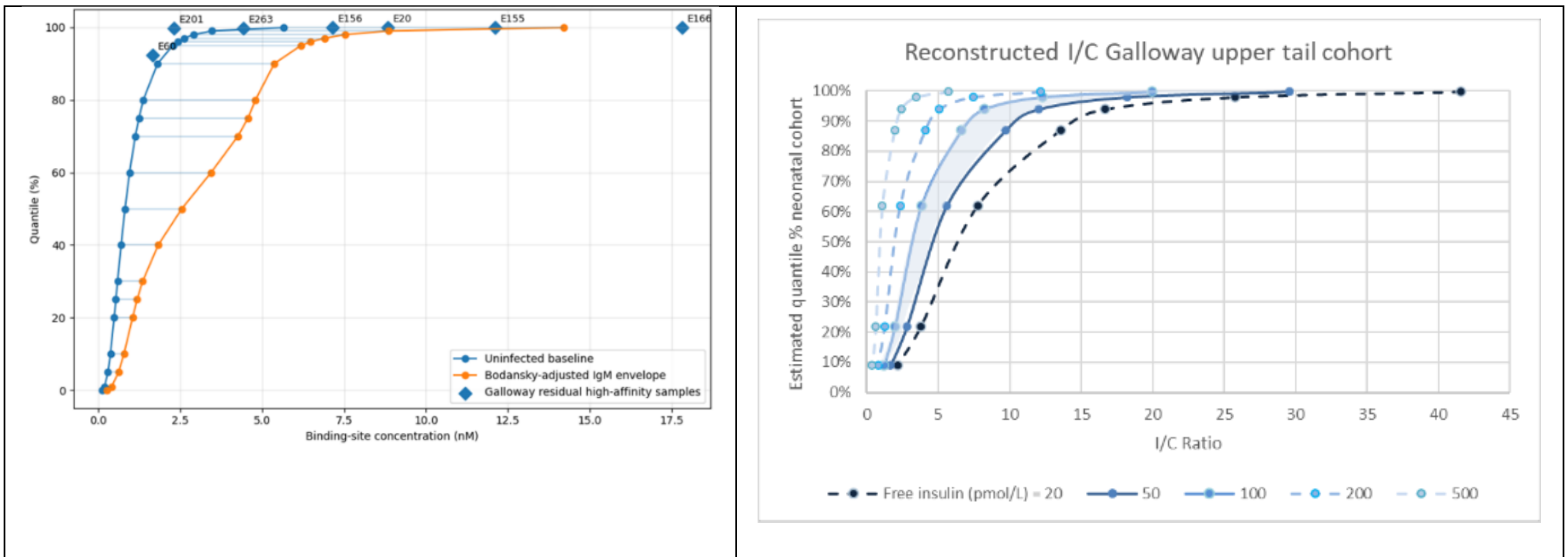

A. Infection-associated amplification shifts neonatal binding-site distribution upward by approximately threefold. Galloway [16] high-binding samples extend into the upper tail of the infection-response envelope.	B. Reconstructed infection-associated binding-site concentrations generate markedly elevated I/C ratios across physiologically plausible neonatal free insulin concentrations (median 50 – 100 pmol/L, blue shading).

Figure A2: Infection-associated amplification of neonatal insulin binding. Infection extends the upper tail of the baseline concentration distribution and generates markedly elevated reconstructed I/C ratios.

3. Consolidation

Four independent lines of evidence converge to constrain the dominant baseline neonatal insulin-binding activity in a non-infected cohort to high affinity. (KD <= 1e-10 M).

Direct ligand-excess calibration together with independent reconstruction from I/C measurements, early-onset type one diabetes comparison and physiological limits from free insulin measurements constrain binding site concentration to the low nanomolar regime operating up to maximum concentration range of 5 – 6 nM in the upper tail (**Figure A3**). Binding site scale is expressed in nM_{HA} units and can be transformed to equivalent lower affinity concentrations using standard equilibrium occupancy relationships.

Threefold infection-associated amplification over the high-affinity baseline as reported in [16] is consistent with direct measurements from Bodansky et al [38] and further showed strong evidence of complexing. Latticing of high affinity component (possibly IgM)] may act to raise effective affinity (avidity) above measured values of KD_{eff} ~5e-11 M, consistent with established polyvalent IgM behaviour [79,81-84]**.**

In the absence of firm characterisation, a parallel possibility of weaker affinity binding across a polyclonal distribution or a high concentration of an unidentified lower affinity protein cannot be ruled out. Although the molecular identity of the baseline neonatal binding agent remains unresolved, the quantitative affinity and concentration envelope in **Figure A3** can be applied independently of binding species by adjusting molar binding-site concentration for affinity.

Finally, **Figure A3**, the result of this annex, repeats **Figure 2B** in the main manuscript. Further details on this derivation can be requested from the authors.

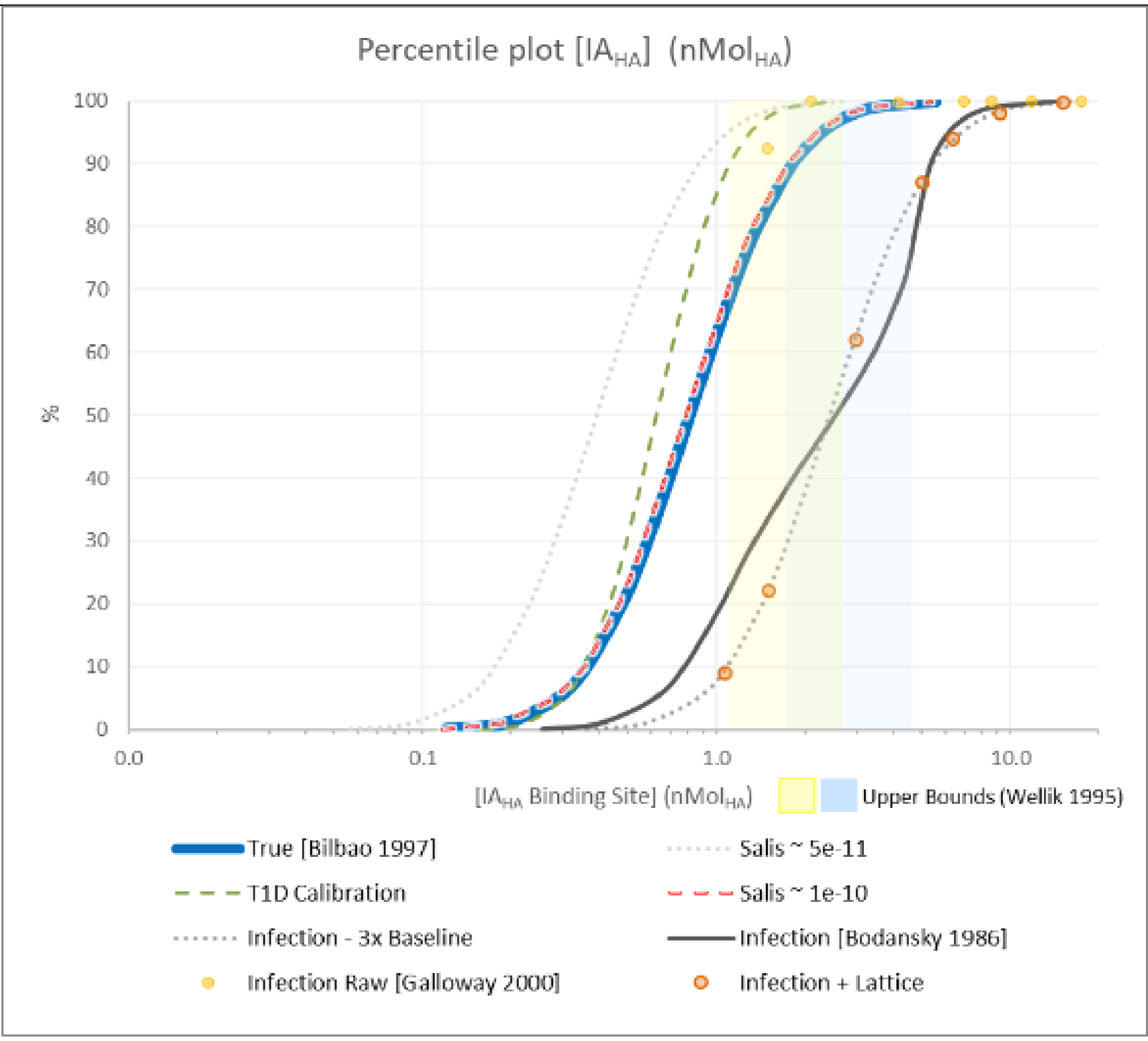

Consolidated neonatal insulin-binding concentration envelope derived from independent affinity and concentration constraints. Baseline distributions derived from ligand-excess calibration are independently corroborated by I/C reconstruction, early-onset T1D comparison and free-insulin measurements. Infection-associated amplification extends the upper tail into the low 10s nM range. Observed high-binding neonatal samples [16] (gold points) lie within or near the upper tail. The other references in the legend are to Bilbao et al [15]and Galloway et al [16] and Salis et al [5,6].

Figure A3: Consolidated neonatal insulin-binding concentration profile including infection-associated amplification repeating main result in the body of the article, matching the result presented in **Figure 2B** in the main manuscript.

References used only in Appendix-A: Which are also listed in main article reference list

74. Vardi, P., Dib, S.A., Tuttleman, M., Connelly, J.E., Grinbergs, M., Radizabeh, A., Riley, W.J., Maclaren, N.K., Eisenbarth, G.S., and Soeldner, J.S., Competitive insulin autoantibody assay: prospective evaluation of subjects at high risk for development of type I diabetes mellitus. Diabetes, 1987. 36(11): p. 1286-1291.

75. Hämäläinen, A.M., Ilonen, J., Simell, O., Savola, K., Kulmala, P., Kupila, A., Simell, T., Erkkola, R., Koskela, P., and Knip, M., Prevalence and fate of type 1 diabetes-associated autoantibodies in cord blood samples from newborn infants of non-diabetic mothers. Diabetes/metabolism research and reviews, 2002. 18(1): p. 57-63.

76. Stanley, H.M., Norris, J.M., Barriga, K., Hoffman, M., Yu, L., Miao, D., Erlich, H.A., Eisenbarth, G.S., and Rewers, M., Is presence of islet autoantibodies at birth associated with development of persistent islet autoimmunity? The Diabetes Autoimmunity Study in the Young (DAISY). Diabetes care, 2004. 27(2): p. 497-502.

77. Lindberg, B., Ivarsson, S.-A., Landin-Olsson, M., Sundkvist, G., Svanberg, L., and Lernmark, Å., Islet autoantibodies in cord blood from children who developed type I (insulin-dependent) diabetes mellitus before 15 years of age. Diabetologia, 1999. 42(2): p. 181-187.

78. Naserke, H., Dozio, N., Ziegler, A.-G., and Bonifacio, E., Comparison of a novel micro-assay for insulin autoantibodies with the conventional radiobinding assay. Diabetologia, 1998. 41(6): p. 681-683.

79. Keyt, B.A., Baliga, R., Sinclair, A.M., Carroll, S.F., and Peterson, M.S., Structure, function, and therapeutic use of IgM antibodies. Antibodies, 2020. 9(4): p. 53.

80. Trabucchi, A., Iacono, R.F., Guerra, L.L., Faccinetti, N.I., Krochik, A.G., Arriazu, M.C., Poskus, E., and Valdez, S.N., Characterization of insulin antibodies by Surface Plasmon Resonance in two clinical cases: brittle diabetes and insulin autoimmune syndrome. PLoS One, 2013. 8(12): p. e84099.

81. Goldstein, B., Theory of hapten binding to IgM: The question of repulsive interactions between binding sites. Biophysical Chemistry, 1975. 3(4): p. 363-367.

82. Marrack, J.R., The Chemistry of Antigens and Antibodies. 1938.

83. Dembo, M. and Goldstein, B., Theory of equilibrium binding of symmetric bivalent haptens to cell surface antibody: application to histamine release from basophils. The Journal of Immunology, 1978. 121(1): p. 345-353.

84. Oostindie, S.C., Lazar, G.A., Schuurman, J., and Parren, P.W., Avidity in antibody effector functions and biotherapeutic drug design. Nature Reviews Drug Discovery, 2022. 21(10): p. 715-735.
